# Demographic Effects of Habitat Restoration for the Grey-Crowned Babbler *Pomatostomus temporalis*, in Victoria, Australia

**DOI:** 10.1371/journal.pone.0130153

**Published:** 2015-07-15

**Authors:** Peter A. Vesk, Doug Robinson, Rodney van der Ree, Caroline M. Wilson, Shirley Saywell, Michael A. McCarthy

**Affiliations:** 1 Australian Research Council Centre of Excellence for Environmental Decisions, School of BioSciences, The University of Melbourne, Parkville, Victoria, Australia; 2 Trust for Nature, Melbourne, Victoria, Australia; 3 Australian Research Centre for Urban Ecology, c/, School of BioSciences, The University of Melbourne, Parkville, Victoria, Australia; 4 Friends of the Grey-crowned Babbler, Euroa, Victoria, Australia; University of Sydney, AUSTRALIA

## Abstract

**Background:**

Considerable resources are spent on habitat restoration across the globe to counter the impacts of habitat loss and degradation on wildlife populations. But, because of time and resourcing constraints on many conservation programs, the effectiveness of these habitat restoration programs in achieving their long-term goals of improving the population viability of particular wildlife species is rarely assessed and many restoration programs cannot demonstrate their effectiveness. Without such demonstration, and in particular demonstrating the causal relationships between habitat restoration actions and demographic responses of the target species, investments in restoration to achieve population outcomes are of uncertain value.

**Approach:**

Here, we describe an approach that builds on population data collected for a threatened Australian bird – the Grey-crowned Babbler *Pomatostomus temporalis* - to evaluate how effectively targeted habitat restoration work improves its viability. We built upon an extensive historical survey by conducting surveys 13 years later at 117 sites stratified by presence/absence of restoration works and by detection or not of birds in the first survey. Our performance metric was the number of individuals in a social group, which is both a measure of local abundance and directly related to breeding success. We employed an occupancy model to estimate the response of Grey-crowned Babbler social group size to the effects of time, restoration works, local habitat as measured by the density of large trees, and distance to the nearest other known group of babblers.

**Results and implications:**

Babbler group size decreased over the survey period at sites without restoration works, but restoration works were effective in stemming declines where they were done. Restoration was responsible for a difference of about one bird per group of 3-5 individuals; this is an important effect on the reproductive success of the social group. Effectiveness of restoration works targeted at the Grey-crowned Babbler was only demonstrable by sampling through time and including control sites without restoration works. This work demonstrates that while calls for better monitoring of restoration are valid, scope exists to recover a signal of effectiveness from opportunistic retrospective analyses.

## Introduction

Loss, fragmentation and degradation of habitat threatens the conservation of biodiversity across the globe [1]. Halting land clearing and restoring and replacing vegetation that has been degraded or cleared are the main approaches to prevent and reverse this loss of biodiversity [2,3]. However, despite a widespread global push for ecological restoration as a means of reversing species’ declines, case studies demonstrating long-term demographic responses to targeted habitat restoration remain scarce [4].

The difficulty of demonstrating the environmental benefits of expenditure on vegetation restoration and other management actions has raised concerns in Australia, the UK and USA [5]. In Australia, for instance, the National Audit Office has consistently highlighted an inability of projects to demonstrate the environmental benefits of expenditure on vegetation restoration and other management actions in relation to longer-term biodiversity outcomes such as positive population responses [6,7]. Demonstrating the environmental benefits of vegetation management to populations of target species is difficult for many reasons, including insufficient monitoring data, potentially long time frames over which responses will likely occur, an absence of data prior to the vegetation management actions being implemented, and difficulties of detecting changes against a changing background [8,9,10].

While suggestions about a priori monitoring requirements are available [10], these will not help us to evaluate species responses to restoration activities under the common project setting where restoration activities have already been implemented without tailored monitoring programs. Moreover, until very recently, at least in Australia, restoration works in targeted conservation programs have rarely been implemented synchronously with any monitoring program because of time and project funding constraints [8]. In practice, budget, human and physical resources often mean that restoration works are carried out through time according to short-term conservation priorities, which may themselves change. Restoration work is (sensibly) targeted at sites where it is expected to yield greatest benefit. Hence, in a species conservation setting, sites may be prioritised for restoration based on the occupancy of a target species. Such adaptive management complicates assessment in at least two ways; restoration works are not randomly allocated to sites, nor are the treatments applied homogeneously across sites. Nevertheless, researchers, managers and the wider public need to understand the effectiveness of such conservation programs despite the shortcomings of the initial monitoring design and implementation of restoration. While some work has considered how to make use of legacy datasets not intended for monitoring [11], more could be done to assess the many existing vegetation management and restoration programs whose effectiveness remains undemonstrated.

Here we evaluate the effectiveness of a restoration program implemented to improve the viability of a target species when the original restoration works were not established in a controlled, experimental design but were targeted at key sites where landholders agreed to undertake the restoration works. Accordingly, we are addressing issues of assessing demographic outcomes with suboptimal original data, non-random and heterogeneous management application. We stress that this reflects the reality of numerous restoration projects worldwide and thus the importance of understanding such projects.

Using restoration works for the Grey-crowned Babbler (*Pomatostomus temporalis*) in northern Victoria, Australia as an example, we show that the benefits of vegetation management can be quantified, providing evidence of the magnitude of those benefits and highlighting scope for further evaluation. One oft-cited difficulty for monitoring and evaluating restoration is the absence of a clear objective [12,13]. In the current case study, this was not a problem; the objective was to change the population trend of Grey-crowned Babblers in the study region from a decline into an increase, thus providing an example of a targeted program that should be possible to evaluate. The problem is apparently simple, yet the pathway to robust analysis is not.

An important performance measure of restored vegetation and habitat is the use and recolonisation of restored areas by fauna. Many fauna from a wide range of taxa will use restored habitat [14,15], with birds figuring prominently in studies [4,16] because they can be efficiently surveyed and respond to changes in vegetation at scales similar to those affected by landuse change [17,18]. Woodland birds in Australia use plantings of native trees and shrubs in areas where woodlands have been cleared for agriculture [19,20,21]. Yet use, or occupancy, may not indicate increased population viability [22,23] because breeding success of birds of concern may not be enhanced by replanting activities [24]. Some notable successes in the United Kingdom are programs specifically targeting the habitat and resource needs of the species or guild of concern, e.g. Cirl Bunting (*Emberiza cirlus*) [25], Stone-curlew (*Burhinus oedicnemus*) and Grey Partridge (*Perdix perdix*) [26] and breeding wading birds [27]. The generality of such results is unclear. Understanding and reversing declines in Australian woodland birds must consider how management changes population processes [28].

The Grey-crowned Babbler is a large (23–29 cm long, c. 80g), sedentary, cooperatively breeding species of woodland bird [29,30,31] that was once found throughout much of Australia [32]. However, like many species of woodland birds in Australia [33,34], the Grey-crowned Babbler has declined in extent and abundance through much of its former range due to extensive clearing of woodland for agriculture [35,36]. These declines have been most evident in the southern part of their range, where land clearing has been most intensive. As a consequence, the Grey-crowned Babbler is regionally extinct through much of its former range in south-eastern Australia [35,37].

In response to the rapid decline of this species in the state of Victoria, a habitat restoration program began in 1994, when the estimated Victorian population consisted of only 260 family groups and was still declining [38]. Subsequent studies have suggested the number of babblers and average family group size have increased in the project areas since habitat restoration works began [39,40]. In none of these examples, however, was it possible to demonstrate robust relationships between the restoration works and the demographic or population responses of the species. The aim of this study thus was to quantify the effectiveness of a targeted habitat restoration program aimed at reversing declines in the occupancy and abundance of Grey-crowned Babblers.

## Materials and Methods

### Study Area

The study was conducted in northeastern Victoria, Australia (roughly centred on latitude -36°38’, longitude 145°32’). The area is predominantly cleared agricultural land with about 5–10% tree cover, most of which occurs as remnant strips along roads and streams, with a smaller proportion occurring as scattered individual trees within farmland or small (<5 ha) patches. The dominant species of canopy tree is Grey Box *Eucalyptus microcarpa* with River Red Gum *E*. *camaldulensis* also common. Clearing mainly occurred from the mid 1800s to early 1900s. Most woodland remnants in the study area have been severely degraded by livestock grazing, weed invasion and timber cutting [41]. Surrounding landuses are mainly grazing on either exotic pasture or mixed native and exotic pasture, and cropping, mostly of wheat and canola. The study area (Violet Town, Moglonemby and Molka districts) was chosen because it has been the main focus for Grey-crowned Babbler conservation in Victoria since the early 1990s [39,40]. In the study area, Grey-crowned Babblers are almost entirely restricted to roadside vegetation and adjacent remnants [39].

### Restoration works

Restoration works included the planting of native or exotic trees and shrubs in block or corridor arrangements, natural regeneration of trees and shrubs and fencing to exclude livestock, within or adjacent to remnants and roadsides. The most common type of restoration works, present at most restoration sites, were ‘Buffers’, being a long fenced strip parallel to existing treelines, enhanced by planting and/or natural regeneration. There was usually more than one type of restoration works at each study site, making it difficult to separate the effects of each restoration works type. Therefore, we do not distinguish between different types of restoration works. In this paper, we only use the presence or absence of restoration works. Over the period we consider here, restoration was expected to influence babblers through increases in shrubs and small trees (see below, Habitat and other covariates).

### Grey-crowned Babbler surveys

Grey-crowned Babblers (hereafter referred to as babblers) live in family groups of 2–12 birds (median 4), occupying territories of 2–53 ha [29,36,42]. The number of birds in a family group of babblers is an important indicator of local population status. Breeding success in the species is positively related to group size [29,30,31], because juveniles remain as helpers at the nest for up to four years [42]. The number of helpers increases the number of fledglings [29]. Larger groups are more viable locally and provide more potential dispersers for colonisation of vacant habitat [42]. The larger regional populations in Victoria have larger family groups than the smaller regional populations (D. Robinson, Unpublished Data). Larger babbler groups tend to occupy territories with intermediate (20–50%) woody cover and a mix of vegetation types and structure [42]. Similar relationships between habitat variables and group size and/or breeding success have also been found for other species of Australian babbler [43,44]. These inter-relationships among group size, breeding success and regional populations make group size a relevant metric for Grey-crowned Babbler conservation.

Our key response variable was the group size of babblers in 1995 and 2008 and for a subset of sites, in 2009. Populations of babblers are not expected to show large year-to-year fluctuations owing to their relatively low reproductive output and high survival [36,45]. Initial population data for the study sites came from a systematic survey for babblers across the region between May and August 1995, roughly coinciding with the start of the habitat restoration works that we consider here (1995–2002). These surveys consisted of continuous searches of approximately ten minutes, (lasting up to 20 minutes to confirm group size when necessary), of 1 km sections of roadside habitat, looking for birds or their conspicuous roost nests [46]. If by the midpoint of the survey no birds were encountered, or if nests were evident, but birds were not observed, a 3-minute playback of territorial calls was used. Where no response was elicited with call playback, the site was revisited at a later date and call playback again used. For every babbler group detected, the location, habitat and number of birds in the group was recorded.

Surveys and incidental observations over subsequent years identified a second set of sites at which babblers were not detected in 1995 but were subsequently observed up until 2005. This second set of sites represent apparent colonizations (but possibly also failed detections in 1995) and may be driven by different processes (to persistence). These apparently newly-colonized sites were then targeted for restoration activities (see next section), a second reason to separate them from the original dataset. With these two sets of sites together, we had 229 potential sites consisting of all sites across the study area where babblers were detected between 1995–2005. Of the 229 sites, 57% had restoration works between 1995 and 2002. In May-August 2008, we re-surveyed a stratified, random sample of 117 of these 229 sites after removing sites along major roads (traffic volume >500 vehicles per day). We did not wish for the increased babbler mortality associated with high traffic volumes [47], to confound our estimates. Hence our inferences only apply to populations away from high traffic volume roads. Sites where habitat restoration works were <6 years old (i.e. planted after 2002) were also excluded because plants of this age are generally too small to provide food or nesting habitat for babblers (D. Robinson unpubl. data). Surveys were undertaken with permission of local land managers (Strathbogie Shire and private land owners) and permits and approvals under relevant Animal Care and Ethics Committee were not required to undertake observational bird surveys or habitat assessments.

The 117 sites resurveyed in 2008 were stratified by (i) district; (ii) detection of babblers in 1995 (Set 1, 67 sites) or only subsequently (Set 2, 50 sites) and (iii) presence (or absence) of restoration works. Sites were also randomly selected, attempting to ensure approximately equal number of sites in each combination of these three factors. Preliminary analyses indicated that babbler groups were larger and less likely to decline in the Violet Town district, but similarity in trends and uncertainty in the estimates gave no clear reason to distinguish the districts; we do not refer to analyses that compared districts again. In 2008, the number of birds observed during a 10 minute survey was recorded at each site. If no birds or recent nests were observed, a 3 minute playback of territorial songs and calls was broadcast. In May 2009 a subset of 41 sites, Set 3, were resurveyed using the same method as in 2008 to detect changes in numbers over one breeding season. These sites were a stratified random sample by district from sites known to be occupied in 2008, with 21 with restoration works and 20 without restoration works. Because juvenile babblers stay with their family group for at least one year after birth [42], positive changes in numbers were assumed to represent increases due to breeding success; negative changes were assumed to represent losses due to mortality or dispersal.

Because the 2008 and 2009 surveys were conducted only once at each site, and those in 1995 only visited twice if the first survey resulted in non-detection, we completed a separate round of surveys to estimate the detectability of birds. In June 2010, a series of four counts was conducted at a set of 29 sites using the same method as 2008 and 2009, with the order of sites randomly changed between survey days so that sites were surveyed over a range of times of day. This is called a ‘metapopulation design’ [48,49].

### Habitat and other covariates

The vegetation of the 117 sites surveyed was assessed in 1995 and 2008. Survey sites were 0.25 ha. Roadside sites had a vegetated strip of various length and width with the width typically 20–40 m, the area of road being excluded; all other sites were 50 m x 50 m. The number of woody plants (>1.3 m in height) in three size classes (<20 cm diameter at breast height (dbh), 20–60 cm dbh, >60 cm dbh) were counted in both the site and within a 100 m buffer surrounding the perimeter of each. Small trees ((<20 cm dbh) are often used for roosting and nesting, while large trees are important for foraging for insects [32].

Initial analyses indicated that babbler numbers were positively correlated with densities of both small and large trees. We checked that sites sampled to represent restoration actions over 1995–2002 had similar numbers of small and large trees in 1995 using single factor Generalised Linear Models. If anything, restoration works were carried out on sites with lower density of both small and large trees: log(small tree density) = 3.44–0.113 (± 0.034) * restoration work, p < 0.001, 115 d.f.); log(large tree density) = 3.50–0.212 (± 0.034) * restoration work, p < 0.001, 115 d.f.). We reasoned that restoration should not increase the number of large trees in 2008, as this would require growth rates of 4 cm DBH yr^-1^. GLM confirmed this: log(large tree density) = 3.45–0.203 (± 0.034) * restoration work, p < 0.001, 115 d.f.). Yet restoration should increase the number of small trees, resulting in higher densities associated with the restored sites in 2008, which it did: log(small tree density) = 5.37 + 0.368 (± 0.012) * restoration work, p < 0.001, 115 d.f.). Because the small trees and shrubs could arise from restoration works, their use as an independent measure of habitat quality was compromised. Instead, we used the number of large trees (>60 cm dbh) within the site as a measure of extant habitat quality to complement the effect of habitat restoration works. Because the number of large trees may have changed over between the surveys, we used the density of large trees onsite in 1995 for the 1995 babbler surveys and the density in 2008 for the 2008 and 2009 babbler surveys.

Based on unpublished research into the effects of distance to other babbler groups on likelihood of site occupancy (D. Robinson unpubl. data), [47], we also measured on a GIS the straight-line distance from the mid-point of each site to the nearest known group of babblers (during 2005–2008) to reflect the capacity of babblers to leave or colonize the site.

### Data analyses

We utilized the framework of binomial mixture modeling for occupancy and abundance formalized by Dorazio & Royle [49,50]. We wrote submodels for observation, occupancy and group size, which we outline below. Variants of these models were considered and results from these are presented in S1 File. Our primary data are the count of birds, Y_*ik*_, recorded in a survey of site *i* in year *k* = 1, 2, 3 for each of 1995, 2008 and 2009, respectively.

#### State Processes

We begin by considering site occupancy—whether a survey site *i* in year *k* overlaps the home range of a family group of babblers. Then, given that a group does occupy a site, what is the site-specific abundance, which we consider the group size at that site. We denote true states and data with capitals and parameters to be estimated in lower case. Thus, we write:

$$Z_{ik}{}_{}\sim \text{ Bernoulli}(\Psi_{ik})$$ (1)

$${\text{N}}_{ik}\sim \text{ zero-truncated Poisson}(Z_{ik}\lambda_{ik})$$ (2)

That is, the true occupancy state, *Z*_*ik*_, depends on the probability of occupancy by a group ψ. The true number of individuals in the family group that uses the site, N, depends on the mean abundance λ, which is truncated to non-zero counts.

We then can write submodels for each of occupancy and group size, which we detail below.

#### Observation processes

Two observation processes were modeled, the first being the detection of a group during a site survey, given that their home range includes the site. This group detection conflates failing to observe the group of birds during a survey when present at the site, with the birds’ use of a wider territory so that the birds may not be available at the site during the survey. This latter process is sometimes called ‘temporary emigration’, and distinguishes a geographically ‘open’ population from one that is ‘closed’ [48]. Chandler et al. [51] present a model for geographically open populations, and our model only differs in that we consider the whole group to be available or not, as opposed to individual birds. The babblers’ large body size, conspicuous behaviour including frequent, loud calls [36], coupled with the generally open habitats, suggest that counts of zero from sites included in babbler home ranges are dominated by the babblers being unavailable during a survey rather than observers failing to detect all birds of the group when they are in fact present at the survey site. Therefore we consider this process the availability of the group to be surveyed conditional upon the site being within the group’s home range. We only allow for false negatives, i.e. missed birds, and not false positives—double counting or misidentification seems unlikely for this distinctive species. Thus, we write:

$$A_{ik}\sim \text{ Bernoulli}\left(pZ_{ik}\right)$$ (3)

$${\text{Y}}_{ik}\sim \text{ Binomial}(dA_{ik},\;N_{ik})$$ (4)

That is, the true state of availability, *A*, of the group for detection depends on the probability that a group is available to be surveyed, *p*. The number of birds observed, Y, depends on the probability of detecting an individual bird, *d*.

Because of the design of the data set (some sites known to be originally occupied, some sites known to have had no babblers detected in two surveys in 1995), *A*_*ik*_ could be further specified in particular submodels. For Set 1, the 67 sites at which babblers had been detected and were thus known to be occupied originally (*Z*_*ik*_ = 1), a group must have been available during the initial 1995 surveys, therefore *A*_i1_ = 1. In 2009, surveys were targeted to estimate the changes in local group size over a single breeding season and restricted to sites where birds were observed, therefore *A*_i3_ = 1.

As mentioned earlier, the data to enable estimation of the individual detection probability *d* and the group availability *p* came from a series of four surveys in June 2010 (i.e., after our primary surveys of 2008 and 2009). These data were modelled as above for the state and observation process except the repetition in sampling is within a season rather than across years, so index *k* becomes *j*.

#### Occupancy submodels

For Set 1, the sites at which birds had been detected in the past, occupancy in 1995 was 1 by definition: *Z*_*i*1_ = 1. For these 67 sites we could write a model for the probability of occupancy in the 2008 surveys, ψ_*i2*_, which amounts to an analysis of changing occupancy:

$$\psi_{i2}=logit^{-1}(c+\gamma_{1}\times dist_{i}+\gamma_{2}\times tree_{i2}+\gamma_{3}\times work_{i})$$ (5)

For Set 2, the 50 apparently, newly-colonized sites, the 2008 occupancy was similarly modelled, differing only in the intercept *c*. We did not model occupancy for 2009, as these were targeted surveys at sites known to be occupied, *Z*_*i*3_ = 1.

#### Group size submodels

We wrote log-linear models for the variation in the mean group size across sites λ_*ik*_. Separate submodels were written for the group size in 1995, 2008 and 2009 surveys with the submodel for 2008 including terms for the effect of time and its interaction with restoration works. The number of large trees varied between the 1995 and 2008, which we did not wish to influence the estimated coefficient, hence requiring the separate submodels. We included a site random effect, *ε*_*i*_, which accounts for the dependencies between group size estimates through time for any given site. Hence, the submodels for the group size at Set 1 sites (in which babblers were detected in 1995) were:

$$\lambda_{i1}=exp(\alpha +\beta_{1}\times dist_{i}+\beta_{2}\times tree_{i1}+\beta_{5}\times work_{i}+\varepsilon_{i})$$ (6a)

$$\lambda_{i2}=exp(\alpha +\beta_{1}\times dist_{i}+\beta_{2}\times tree_{i2}+\beta_{3}+(\beta_{4}+\beta_{5})\times work_{i}+\varepsilon_{i})$$ (6b)

$$\lambda_{i3}=exp(\alpha +\beta_{1}\times dist_{i}+\beta_{2}\times tree_{i2}+\beta_{3}+(\beta_{4}+\beta_{5}+\beta_{6})\times work_{i}+\beta_{7}+\varepsilon_{i})$$ (6c)

where *α* is the intercept and represents the average log_e_-transformed group size in 1995. *β*_1_ is the regression coefficient for the effect of the distance to the nearest other detected group of babblers. *β*_2_ is the regression coefficient for the effect of large trees. *β*_3_ is the regression coefficient for the effect of time between 1995–2008, such that the average (log_*e*_-transformed) group size in 2008 at sites without restoration works would be equal to *α* + *β*_3_. *β*_4_ is the regression coefficient for the effect of change over 1995–2008 due to restoration works. Because practitioners may have conducted habitat restoration at sites that may have been better to start with and to support more birds, we checked for pre-existing differences: *β*_5_ is the regression coefficient for the effect of the presence of restoration works and accounts for any difference at the initial surveys between sites with and without restoration works. *β*_6_ is the regression coefficient for the effect of change over 2008–2009 due to restoration works. *β*_7_ is the regression coefficient for the effect of time between 2008–2009. The only difference for Set 2 sites (where babblers were not detected in 1995) was the omission of a model for 1995 group size.

Set 3 sites (those sampled for the first time in 2008 and 2009) had slightly different submodels for group size as we had no covariate information other than the presence or absence of restoration works:

$$\lambda_{i2}=exp(\alpha +\beta_{3}+(\beta_{4}+\beta_{5})\times work_{i}+\varepsilon_{i})$$ (7a)

$$\lambda_{i3}=exp(ln\lambda_{i2}+\beta_{6}\times work_{i}+\beta_{7})$$ (7b)

It turned out that the difference between the sites targeted for restoration was very close to zero and very uncertain (*β*_5_ = -0.028 ± 0.137, mean ± SE), so we removed this term, resulting in the simpler models, which we report hereafter (though the more complex model is presented in Supplementary Results). Hence, for Set 1:

$$\lambda_{i1}=exp(\alpha +\beta_{1}\times dist_{i}+\beta_{2}\times tree_{i1}+\varepsilon_{i})$$ (8a)

For Sets 1 & 2:

$$\lambda_{i2}=exp(\alpha +\beta_{1}\times dist_{i}+\beta_{2}\times tree_{i2}+\beta_{3}+\beta_{4}\times work_{i}+\varepsilon_{i})$$ (8b)

$$\lambda_{i3}=exp(\alpha +\beta_{1}\times dist_{i}+\beta_{2}\times tree_{i2}+\beta_{3}+(\beta_{4}+\beta_{6})\times work_{i}+\beta_{7}+\varepsilon_{i})$$ (8c)

and for Set 3:

$$\lambda_{i2}=exp(\alpha +\beta_{3}+\beta_{4}\times work_{i}+\varepsilon_{i})$$ (8d)

$$\lambda_{i3}=exp(ln\lambda_{i2}+\beta_{6}\times work_{i}+\beta_{7})$$ (8e)

We used Bayesian inference and all models were fitted with MCMC methods using JAGS v3.2.0 [52] run through R v2.15.0 via the package R2jags v0.03–08 [53]. In all cases we ran three chains and checked for convergence visually and by using the potential scale reduction factor [54]. Finding that 2 000 samples was sufficient as a burn-in, these were discarded and inference based on the next 100 000 samples. We used vague, normally-distributed priors with mean 0.0 and standard deviation 100 for all regression coefficients, except for the occupancy submodel of sites where Babblers were detected in 1995. In that case we used weakly informative priors to aid convergence. For the intercept we used a Cauchy prior with mean 0.0 and scale 10, for the regression coefficients we used a Cauchy prior with mean 0.0 and scale 2.5 [55]. We centered the covariates and scaled them by dividing by twice the standard deviation. This makes the effect sizes based on the estimated coefficients of binary and continuous variables comparable [55]. The site random effect was modelled as normally distributed with a mean zero and standard deviation to be estimated from the data, with a Uniform(0,10) prior. Code is in S2 File.

## Results

### Observed counts

**Fig 1:**
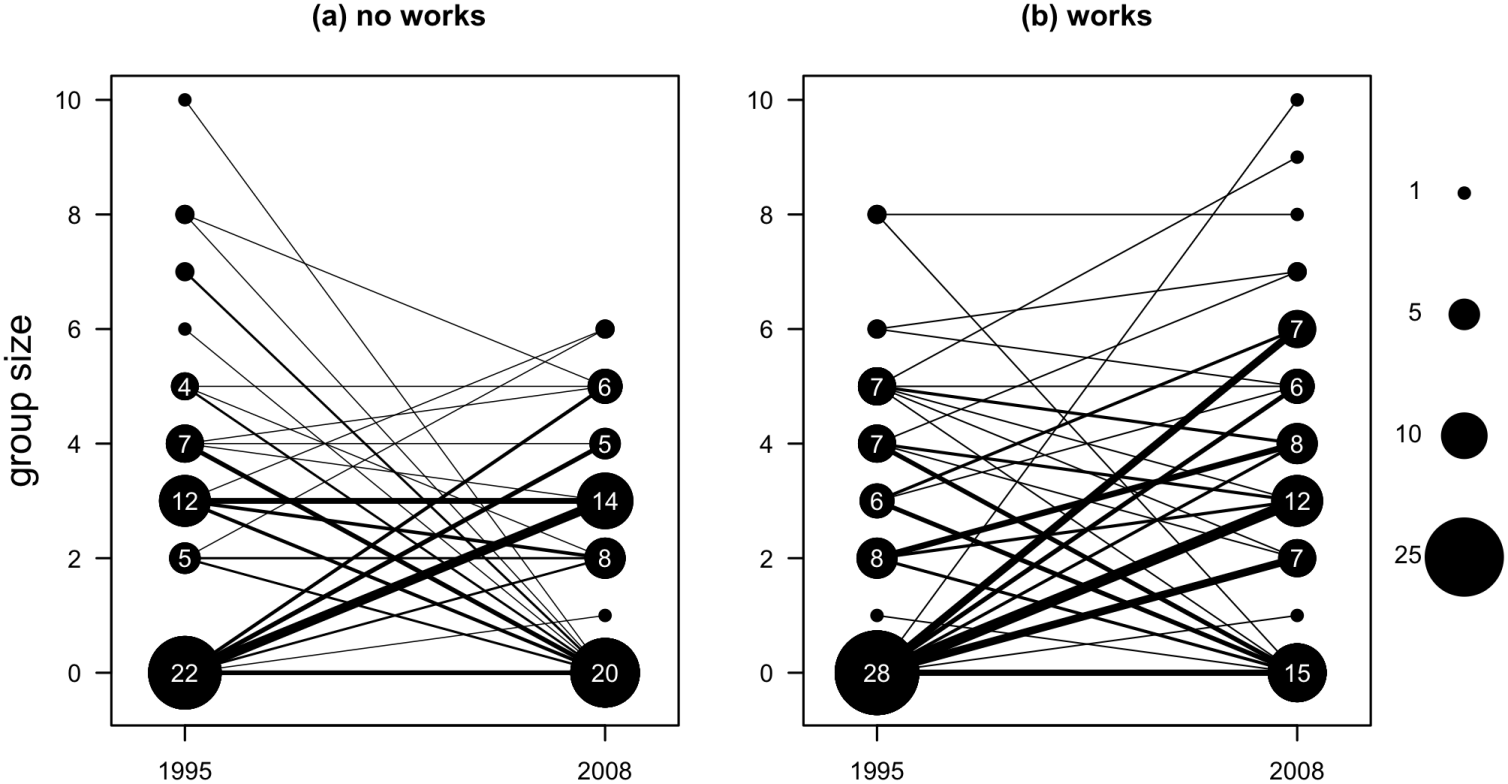
Observed counts of Grey-crowned Babblers in sites surveyed in 1995 and 2008. (a) sites without restoration works; (b) sites with restoration works. Counts of zero can represent true absence or failed detections. Symbol size and number within denote frequency of that group size within the sampling period, according to the design and, not wider occupancy rates. Lines connect the two observations in time at particular sites. Line widths reflect the frequency of that pairing; the thickest line represents eight sites.

Grey-crowned Babblers were detected at 58% and 74% of the 117 sites in 1995 and 2008, respectively. Observed group sizes during all surveys ranged from one to ten birds (Fig 1). Observed counts suggest that at sites with restoration works, fewer sites yielded counts of zero in 2008, and more groups increased in numbers, than at sites without restoration works (Fig 1).

**Fig 2:**
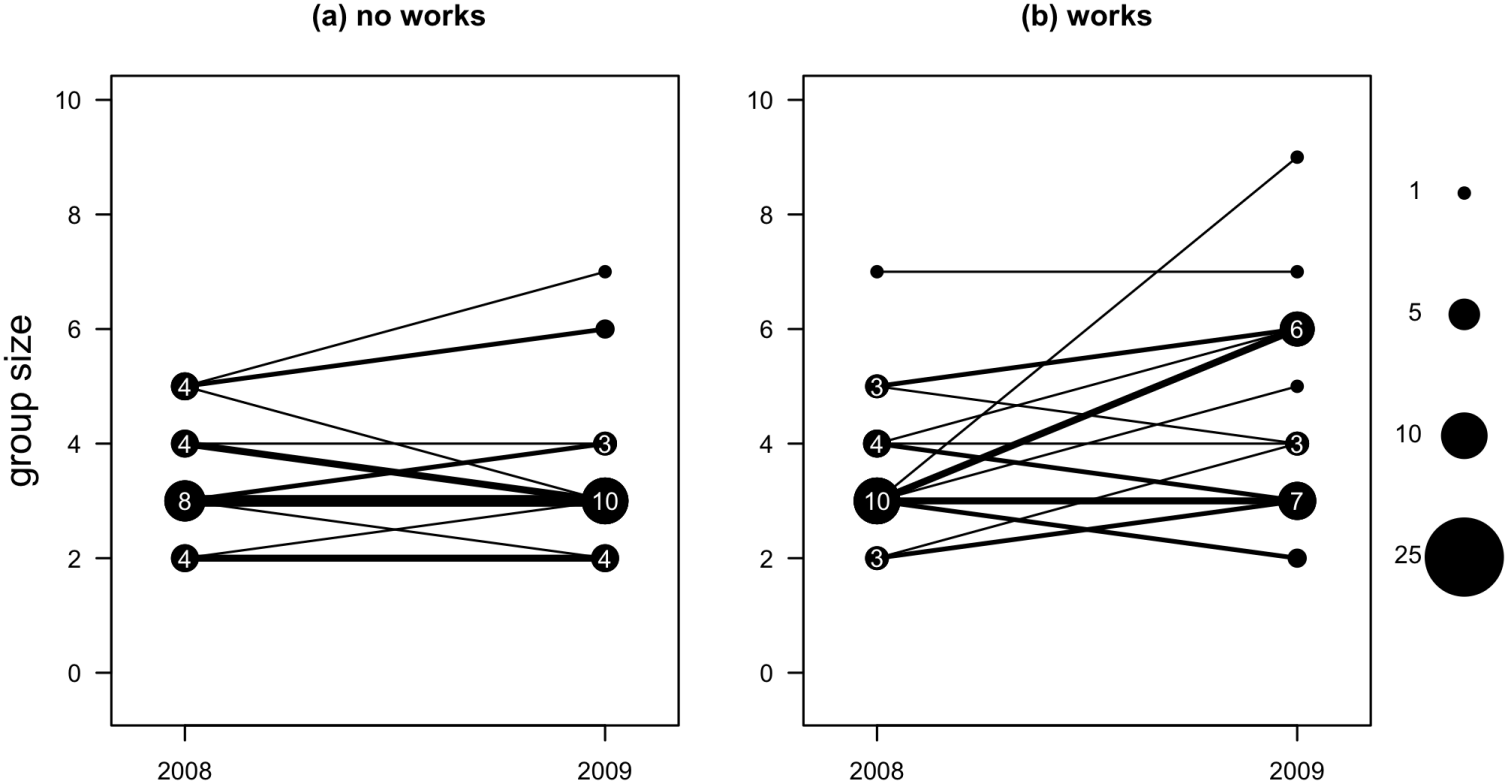
Observed group size of babblers in a subset of sites surveyed in 2008 and resurveyed in 2009. (a) Sites without restoration works; (b) sites with restoration works. The symbol size, and numbers within, reflect frequency of that group size. Lines connect the two observations in time at particular sites. Line widths reflect the frequency of that pairing.

To address the consistency of effects over a single subsequent breeding season, we compared a subset of 41 sites in 2008 and 2009 (Fig 2). For sites without restoration works, increases and decreases of group sizes were of similar frequency and magnitude. For sites with restoration works, decreases were about as common as, and of similar magnitude to, sites lacking restoration works. However, increases appeared more common and of greater magnitude in sites with restoration works than in sites without restoration works.

Models with site random effects had lower deviance than models without (S1 Table, S1 File). Here we focus on model M2, which incorporated a site-level random effect, but no starting differences between restored and unrestored sites, and resulted in the lowest deviance of the models we considered (S1 Table, S1 File). The Supplementary Results (S1 File, S1, S2 and S3 Figs) present inference for a variety of models representing different assumptions about the processes driving occupancy and abundance.

### Occupancy

**Fig 3:**
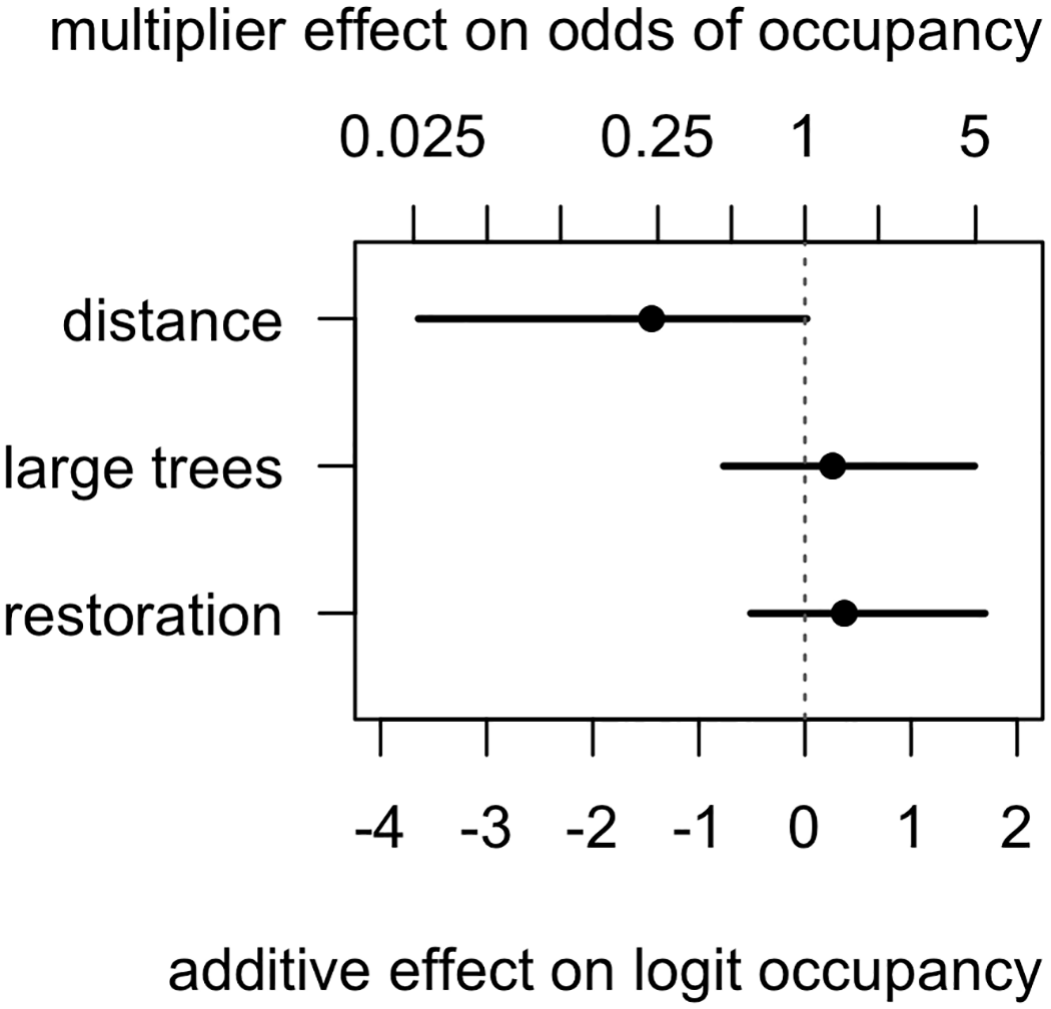
Parameter estimates from the occupancy submodel of Grey-crowned Babblers for sites at which babblers were detected in 1995. Symbols represent point estimates as the posterior median (and 95% credible intervals represented as lines). The x-axis presents the effect size, which is on a logit scale, the second x-axis, above the graph, presents the effects transformed back into odds ratios (or odds for the intercept). The parameter estimates for the various effects represent the additive change to the mean probability of occupancy resulting from the observed range for the particular factor. Interpretation of effects on the raw scale is as the multiplicative change to the raw mean probability of occupancy.

Not all sites that were known to be occupied in 1995, i.e. Set 1, remained occupied in 2008 (Fig 1). The average probability of occupancy at these sites without restoration work, with the average large tree density and average distance from known occupied site was 0.605 (hereafter 95% credible intervals appear in brackets thus: (0.453, 0.801)). The farther the nearest known group of babblers, the less likely a given site would remain occupied by babblers in 2008 (Fig 3). Across the range of distances observed, this accounted for about a fourfold change in the odds of occupancy. In other words, sites were less likely to remain occupied by a group of babblers if there were no nearby groups. Neither the density of large trees nor restoration works had clear effects on occupancy, though their point estimates were positive (Fig 3). When we fitted the linear model for occupancy to Set 2 sites in 2008 regression coefficients were little changed (S1 Fig). However, average occupancy for Set 2 sites without restoration work, with the average large tree density and average distance from known occupied site was much higher than in Set 1: 0.986 (0.994, 1.000).

### Group size

**Fig 4:**
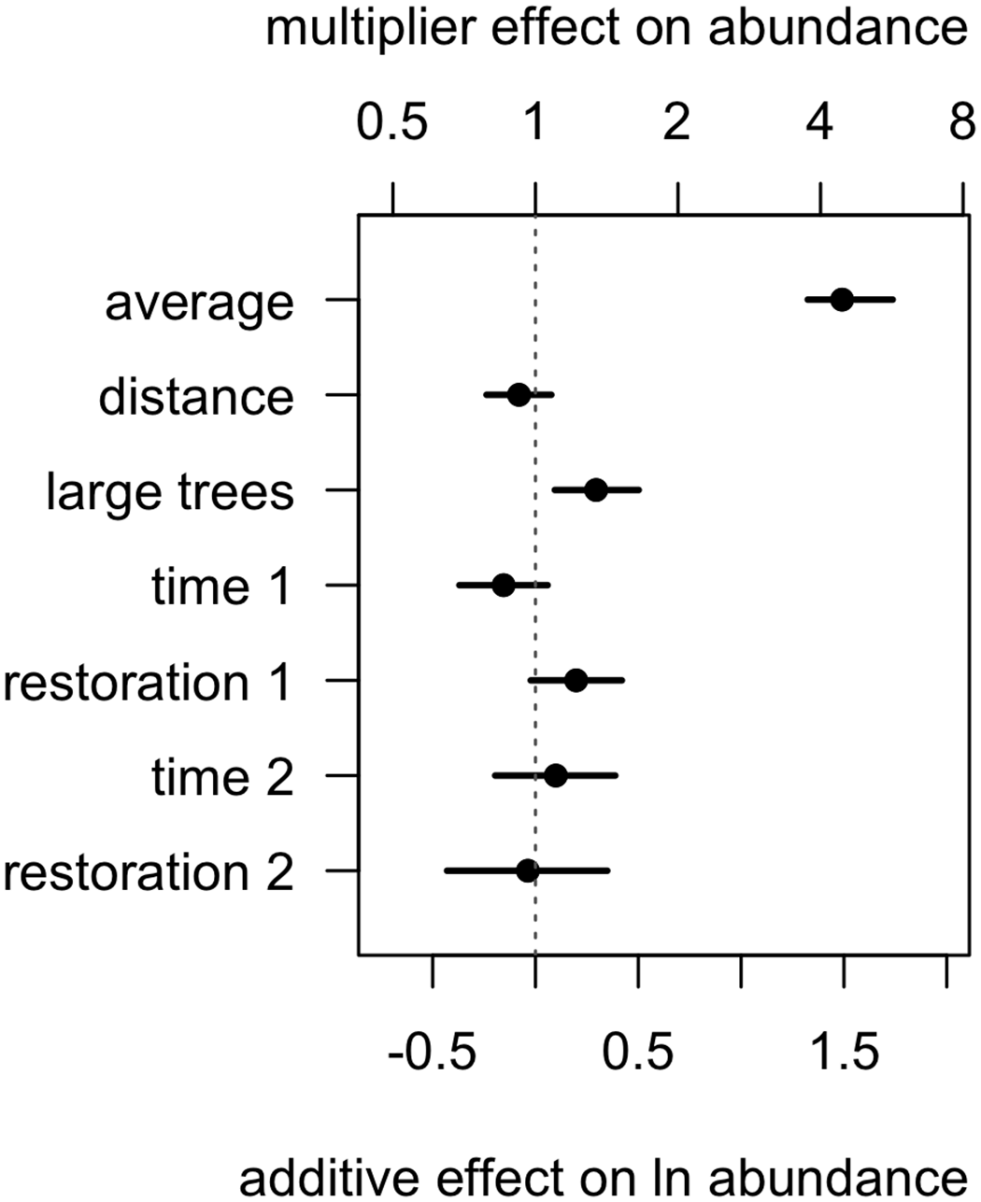
Parameter estimates from the group size submodel of Grey-crowned Babblers, conditional upon occupancy at a site. Symbols represent point estimates as the posterior median (and 95% credible intervals represented as lines). The x-axis presents the effect size, which is on a log scale, the second x-axis, above the graph, presents the effects transformed back into raw numbers. The ‘average’ is the intercept and is the mean group size in 1995 for the average site, at the average distance from nearest group and with the average density of large trees. The parameter estimates for the various effects represent the additive change to the log(mean group size) resulting from the observed range for the particular factor. Interpretation of effects on the raw scale is as the multiplicative change to the raw mean group size.

Of our covariates, large trees had the greatest positive effect on babbler group size (Fig 4). Larger groups occurred where the density of large trees was higher. Babbler group size declined ~15% between 1995 and 2008 at sites without restoration. The presence of restoration works was associated with about 22% larger groups in 2008, effectively offsetting the reduction through time. There was a possible small, but uncertain effect of distance, such that the further groups were from the next nearest group, the smaller the group size.

The estimated average change over the single breeding season (2008–2009) was effectively zero and the effect of restoration works was negligible (Fig 4).

### Modelled changes to group size at occupied sites

**Fig 5:**
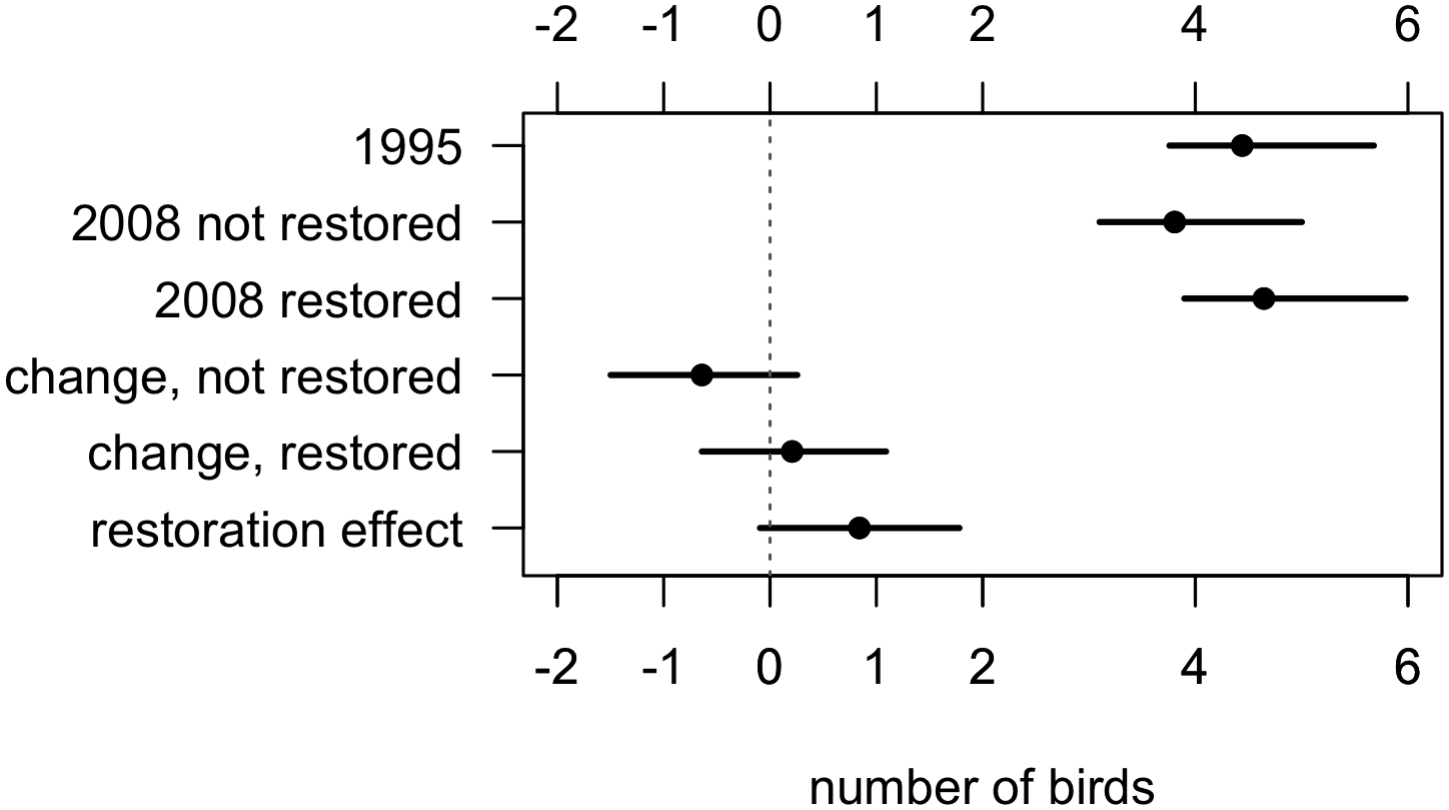
Expected group size for Grey-crowned Babblers at average sites for restored and unrestored sites in 1995 and 2008, the change in group size over that period and the effect of restoration on that change.

The mean group size at occupied sites in 1995 was 4.5 (3.8, 5.5) (Fig 5). In 2008 at sites that had not been restored, this had fallen by 0.6 birds (-1.5, 0.3) to 3.9 (3.1, 5.0). By contrast, sites that were restored had babbler groups averaging 4.6 birds (3.9, 6.0) in 2008. The difference in the change between 1995 to 2008 due to restoration was on average 0.8 bird per group (-0.1, 1.8).

## Discussion

The data presented in this study from two primary surveys separated by 13 years demonstrate that the average number of birds in babbler social groups has declined without restoration, but habitat restoration works have stemmed declines at sites where they have been carried out. Because we have sampled through time, included controls, accounted for potential pre-existing differences, we assert that habitat restoration has been effective locally.

### Evaluation of restoration

Had we not sampled controls, we would have concluded that there was no change in the numbers of babblers and been ignorant of the fact that this was a good outcome against an ongoing decline at unmanaged sites. Had we not sampled through time, we would not know that there is ongoing decline and hence perhaps overestimated the effectiveness of restoration works. Admittedly our two major surveys were 13 years apart, but subsequent surveys one year later demonstrate relatively modest year-to-year fluctuations. We caution against inferences about the long-term (i.e. > 14 years) rates of change. Yet we are confident that these surveys reveal trends rather than simply year-to-year fluctuation, as the population dynamics of this species are moderate—owing to the long lifespan and moderate fecundity, but high juvenile survival of the species [29,36].

There are a number of attributes of this case study that enabled our retrospective analysis: first the existence of an extensive survey prior to or contemporaneous with the start of restoration activity; second a sufficient number of sites to enable stratification; and third, available covariate information from the original surveys.

We acknowledge that surveys were conducted somewhat differently in 1995 and 2008, primarily shorter surveys in 2008 and different observers. Yet we feel that this should not overly affect our inferences about the beneficial effects of restoration, primarily because any bias in group-size estimation between the two periods would need to systematically interact with restoration activities. If the second surveys underestimated group size relative to the first surveys, then both sites with and without restoration works would be underestimated to the same degree and the effect of restoration works would not be affected. To overestimate the effects of restoration works, the second surveys would need to be systematically worse at counting birds in unrestored sites than in restored sites. To underestimate the effect of restoration works would require that the second round of surveys were systematically better at counting birds in unrestored sites than restored sites. This latter scenario is at least plausible if restoration made it harder (through denser vegetation, perhaps) to sight and count birds. This scenario would mean that we underestimate the effectiveness of restoration works, which does not negate our general conclusion that restoration works have been effective in stemming local declines.

### Population processes and restoration effort

A recent review of progress on understanding declines in Australian woodland birds highlighted a need to focus on population processes in relation to management [28] and our study has made steps in this direction. The results suggest that widening existing roadside habitat through fencing buffers on the adjacent private land thus excluding livestock, together with additional replanting of native trees and shrubs is an effective way to increase babbler group size. As shown for other species of woodland fauna utilizing linear habitats in Australian woodlands [56], it is possible that habitat width may influence site selection by babblers because of the increased availability of habitat and food resources at the local scale and a reduced need to travel far from nests [43]. Yet restoration effectiveness also makes sense in the context of the species’ preference for structurally-diverse, mature woodland habitat [38,47,57]. Habitat buffers adjacent to existing mature woodland build on and extend this existing resource, in contrast to new (isolated) revegetation projects in cleared farmland that may take over 100 years before they provide ideal habitat for this species [58]. Habitat buffers containing active or passive restoration may also provide a complementary nesting resource as babblers tend to nest in large shrubs or small trees [57]. Thus, the effect of restoration works is likely related to improved breeding success at those sites [43], as the increases over 2008/09 suggest. However, these increases may also have been partly due to higher survival rates of group members, site fidelity and/or the immigration of individuals from other groups [31,42]. Our finding that restoration could account for nearly an extra bird per social group is an important effect, because over a season, it means about an extra ½ fledgling per group whose seasonal production is ~2–6 fledglings [29].

### Population status

While the results from this study demonstrate that restoration works have measurably improved the persistence of babbler groups at local scales, we can say rather less about the wider population. Our understanding of population trends is confounded by not knowing how sedentary the groups are [59]. There are two particular issues here. First, we have not mapped individual group territories, so it is possible that our site-based surveys have double-counted some groups. We do not think that this affects our results, as we have interpreted local scale changes. Second, it is possible that some reorganisation of the population is occurring whereby apparent extinctions and colonisations are merely groups moving from one place to another [59]. Our occupancy model suggests that sites are more likely to be vacated over a 13 year period if the nearest babbler group is far away. This could be emigration, or it could indicate a heightened extinction risk in those sites that are distant from occupied sites. This latter explanation is unlikely as we found little effect of distance on group size. One would expect probability of extinction to be mirrored in declining group sizes. A further alternative explanation is that areas that have sparser distributions of babbler groups provide less suitable habitat in some unmeasured way.

While we observed apparent colonisation, we have not estimated colonisation rates, because all sites sampled had observations of birds at some time; we sampled sites stratified by detection (or not) of the birds in the 1995 surveys. Estimating colonisation would help to answer whether habitat restoration can increase occupancy of patches and thus metapopulation capacity [60]. Clearly, further monitoring of the studied sites would also benefit assessment of ongoing effectiveness of restoration. We feel that more can be done in the way of retrospective analyses of restoration effectiveness (see [11]). But we echo the call for well-designed monitoring programs to be built in to vegetation management and restoration programs [10].

## Supporting Information

S1 File Supplementary results. (DOCX)

S2 File JAGS model code and data. (DOCX)

S1 Fig Alternative parameter estimates from the occupancy submodel of Grey-crowned Babblers for sites at which babblers were detected in 1995. Symbols represent point estimates as the posterior median (and 95% credible intervals represented as lines). Alternative models: M2, which included a site random effect (blue triangles); M6, which in addition incorporated the linear model for occupancy for Set 2 in 2008 (red circles); and, M4 which included the possibility of difference between restored and unrestored sites in 1995 (black squares).
The x-axis presents the effect size, which is on a logit scale, the second x-axis, above the graph, presents the effects transformed back into odds ratios (or odds for the intercept). The parameter estimates for the various effects represent the additive change to the mean probability of occupancy resulting from the observed range for the particular factor. Interpretation of effects on the raw scale is as the multiplicative change to the raw mean probability of occupancy.
(TIF)

S2 Fig Alternative parameter estimates from the group size submodel of Grey-crowned Babblers, conditional upon occupancy at a site. Symbols represent point estimates as the posterior median (and 95% credible intervals represented as lines). Alternative models: M2, which included a site random effect (blue triangles); M6, which in addition incorporated the linear model for occupancy for Set 2 in 2008 (red circles); and, M4 which included the possibility of difference between restored and unrestored sites in 1995 (black squares). The x-axis presents the effect size, which is on a log scale, the second x-axis, above the graph, presents the effects transformed back into raw numbers. The ‘average’ is the intercept and is the mean group size in 1995 for the average site, at the average distance from nearest group and with the average density of large trees. The parameter estimates for the various effects represent the additive change to the log(mean group size) resulting from the observed range for the particular factor. Interpretation of effects on the raw scale is as the multiplicative change to the raw mean group size.
(TIF)

S3 Fig Alternative expected group sizes for Grey-crowned Babblers at average sites for restored and unrestored sites in 1995 and 2008, the change in group size over that period and the effect of restoration on that change. Alternative models: M2, which included a site random effect (blue triangles); M6, which in addition incorporated the linear model for occupancy for Set 2 in 2008 (red circles); and, M4 which included the possibility of difference between restored and unrestored sites in 1995 (black squares).
(TIF)

S1 Table Comparison of alternative models with posterior mean and SD of deviance. (DOCX)
