# Supplementary material for: Demographic Effects of Habitat Restoration for the Grey-Crowned Babbler Pomatostomus temporalis, in Victoria, Australia
Source: PLoS One. 2015 Jul 15;10(7):e0130153. doi: 10.1371/journal.pone.0130153 (PMC4503698; doi:10.1371/journal.pone.0130153)
Supplement: S1 File — (DOCX) [file pone.0130153.s001.docx]

**Supporting Information S1: Supplementary results**

We ran multiple models to examine effects of various assumptions. M2 is the model presented in the main text and includes a site random effect. The features we wished to explore are described here:

1. Including an effect of starting differences between the group sizes of babblers in sites without works and those receiving habitat restoration work.
2. Incorporating a site random effect, which accounts for dependencies between group estimates at individual sites through time.
3. Fitting the linear model of covariate effects on occupancy not only to Set 1 but also to Set 2 sites, those where babblers were not detected in 1995.

Models with a site random effect had lower deviance than those without (Table S1). And the model with the lowest deviance had no further modifications. This appears in the main text. The best three of these were then chosen for plotting in these Supplementary Results to visually inspect effects of model choice on parameter estimates. All three of these incorporated a site random effect, which accounted for dependencies between group size estimates through time in a given site. These were: M2, which included a site random effect (blue); M6, which in addition incorporated the linear model for occupancy for Set 2 in 2008 (red); and, M4 which included the possibility of difference between restored and unrestored sites in 1995 (black).

Broadly, the plots of Occupancy (Figure S1), Abundance (Figure S2) and group size predictions (Figure S3) show similar coefficient estimates. Coefficient estimates were more precise for the model only including a site random effect, M2.

Incorporating fitting a linear model of covariate effects on Occupancy in 2008 to both Set 1 and Set 2, model M6, resulted in little change to estimated covariate effects (Figure S1). The effect of distance to nearby groups is smaller and includes zero, but broadly this result supports the interpretation that babbler occupancy is more strongly driven by isolation of groups (which may reflect unmeasured habitat suitability) than by the density of large trees or the restoration work (Figure S1).

The estimated effect of start differences on Group sizes incorporated in model M4 was negligible (Figure S2). Yet the uncertainty in the parameter estimate propagates through to the estimated parameters for the intercept and effect of restoration (Figure S2). The result of this uncertainty is that predicted changes in group size due to restoration are less certain (Figure S3) than for other models, though unchanged in magnitude.
