# Supplementary material for: Demographic Effects of Habitat Restoration for the Grey-Crowned Babbler Pomatostomus temporalis, in Victoria, Australia
Source: PLoS One. 2015 Jul 15;10(7):e0130153. doi: 10.1371/journal.pone.0130153 (PMC4503698; doi:10.1371/journal.pone.0130153)
Supplement: S2 File — (DOCX) [file pone.0130153.s002.docx]

**Supporting Information S2: JAGS code and data for integrated Occupancy/Abundance model.**

cat('model{

#### Detectability model

for (i in 1:29) # loop round the sites

{

for (j in 1:4) # loop round the repeat

# surveys

{

YD[i, j] ~ dbin(DD[i, j], ND[i]) # number of birds detected

DD[i, j] <- d * PD[i, j] # prob of detection of

# individuals = d if birds

# present in survey

PD[i, j] ~ dbern(pp[i]) # is the group available

# for survey? Indicator

} # close survey loop

pp[i] <- p * occ[i] # prob of presence in

# survey

occ[i] ~ dbern(psi) # do birds occupy site?

# Indicator

ND[i] ~ dpois(lambdaD)T(1,) # the group size

} # close site loop

psi ~ dunif(0, 1) # proportion of sites

# occupied

logit(p) <- logit.p # mean prob of presence

# during a survey, given

# they occupy the site

logit.p ~ dnorm(0, 0.0001)

logit(d) <- logit.d # mean prob of detection of

# individuals

logit.d ~ dnorm(0, 0.0001)

lambdaD ~ dunif(1,15) # prior for the group size

# parameter

######

# Variables:

# yT observations for time T

# DT[i] detection prob at a site for time T

# NT[i] true group size at time T

# PT[i] availability to be observed (0,1)

# pgroupT[i] prob of availability (occurrence x Pr(avail))

# d individual detectability (prob)

# p Prob of group availability at an occupied site

# zT occupancy state of a site (0,1) at time T

# piT[1] prob. of occupancy at a site at time T

# ps1T mean prob of occupancy of sites at time T

# Observation models

for (i in 1:67) { # Set 1, the occupied-in-1995 sites

y1[i] ~ dbin(D1[i], N1[i])T(1,)

D1[i] <- d * P1[i]

P1[i] <- 1

y2[i] ~ dbin(D2[i], N2[i])

D2[i] <- d * P2[i]

P2[i] ~ dbern(pgroup2[i])

pgroup2[i] <- p * z2[i]

}

for (i in 68:117) { # Set 2, the "new" sites

y2[i] ~ dbin(D2[i], N2[i])

D2[i] <- d * P2[i]

P2[i] ~ dbern(pgroup2[i])

pgroup2[i] <- p * z2[i]

}

for (i in 118:131) { # Set 3 sites sampled for 09, not before

y2[i] ~ dbin(D2[i], N2[i])T(1,)

D2[i] <- d * P2[i]

P2[i] <- 1 # these selected because birds observed.

}

for (i in 1:18) {

y3[i] ~ dbin(D3[i], N3[i])T(1,) #all surveys detected birds

D3[i] <- d * P3[i]

P3[i] <- 1

}

for (i in 68:76) {

y3[i] ~ dbin(D3[i], N3[i])T(1,) #all surveys detected birds

D3[i] <- d * P3[i]

P3[i] <- 1

}

for (i in 118:131) {

y3[i] ~ dbin(D3[i], N3[i])T(1,) #all surveys detected birds

D3[i] <- d * P3[i]

P3[i] <- 1

}

# Process models for occupancy

for (i in 1:67) { # Set 1, the occupied-in-1995 sites

z2[i] ~ dbern(pi2[i])

logit(pi2[i]) <- (c + bb[1]*nd[i] + bb[2]*nt.60[i]

+ bb[3] * Works.pa[i] )

}

for (i in 68:117) { # Set 2, the new or apparently colonised sites

z2[i] ~ dbern(psi2)

}

# Process models for abundances

for (i in 1:67) { # Set 1, the occcupied in 1995 sites

N1[i] ~ dpois(lambda1[i])T(1,)

lambda1[i] <- exp(a + b[1] * nd[i] + b[2] * np.60[i] + re[i])

N2[i] ~ dpois(lambda2[i])

lambda2[i] <- exp(a + b[1]*nd[i] + b[2]*nt.60[i] + b[3] + bW[1] * Works.pa[i] + re[i] )

}

for (i in 68:117) { # Set 2, the new or apparently colonised sites

N2[i] ~ dpois(lambda2[i])

lambda2[i] <- exp(a + b[1]*nd[i] + b[2]*nt.60[i] + b[3] + bW[1] * Works.pa[i] + re[i] )

}

for (i in 118:131) {# Set 3 sites sampled for 09, not included before

N2[i] ~ dpois(lambda2[i])T(1,)

lambda2[i] <- exp(a + b[3] + bW[1] * Works.pa[i] + re[i])

}

for (i in 1:18) {

N3[i] ~ dpois(lambda3[i])T(1,)

lambda3[i] <- exp(a + b[1]*nd[i] + b[2]*nt.60[i] + b[3]

+ (bW[1] + bW[2]) * Works.pa[i] + b[4] + re[i])

}

for (i in 68:76) {

N3[i] ~ dpois(lambda3[i])T(1,)

lambda3[i] <- exp(a + b[1]*nd[i] + b[2]*nt.60[i] + b[3]

+ (bW[1] + bW[2]) * Works.pa[i] + b[4] + re[i])

}

for (i in 118:131) {

N3[i] ~ dpois(lambda3[i])T(1,)

lambda3[i] <- exp(log(lambda2[i]) + b[4] + bW[2] * Works.pa[i] )

}

for (i in 19:67) {

y3[i] ~ dbin(d, N3[i])

N3[i] ~ dpois(lambda3[i])

lambda3[i] <- exp(a + (bW[1] + bW[2]) * Works.pa[i] + b[3]+ b[4]+ re[i])

}

for (i in 77:117) {

y3[i] ~ dbin(d, N3[i])

N3[i] ~dpois(lambda3[i])

lambda3[i] <- exp(a + (bW[1] + bW[2]) * Works.pa[i] + b[3] + b[4]+ re[i])

}

for (i in 1:131) {

re[i] ~ dnorm(0, sd^-2)

}

sd ~ dunif(0,10)

# Priors for process models

a ~ dnorm(0, 1.0E-6) # intercept for abundance

for (i in 1:2) {

bW[i] ~ dnorm(0, 1.0E-6) # regression coeff for works effect

# on abundance

}

for (i in 1:3) {

b[i] ~ dnorm(0, 1.0E-6) # regression coeffs for abundance

bb[i] ~ dt(0,2.5,1) # regression coeffs for change in

# occupancy

}

b[4] ~ dnorm(0, 1.0E-6) # regression coeffs for abundance

c ~ dt(0,10,1) # intercept for change in occupancy

# mean model for occupancy probability in T2

psi2 ~ dunif(0,1)

## calculating derived parameters

pg <- exp(a) # group size at start

ng.nw <- exp(a + b[3]) # group size at non-works sites

# after time

ng.w <- exp(a + b[3] + bW[1]) # group size expected at

# works sites after time

ch.nw <- ng.nw - pg # change at sites without works

ch.w <- ng.w - pg # change with works

dif.ch <- ch.w - ch.nw # diff b/w change with works and

# without,

}',file=(model.file<-tempfile())) #END MODEL

DATA look like this:

> gcb.dat

$y1

[1] 3 8 3 4 3 4 4 3 2 3 3 4 5 2 5 5 2 4 5 2 4 5 7 10 3 3 3 7 2 2 4 3 5 2 4 3 3

[38] 5 8 6 4 3 4 2 3 2 2 2 3 8 2 6 5 5 6 8 5 5 4 4 3 4 2 3 1 4 3 0 0 0 0 0 0 0

[75] 0 0 0 0 0 0 0 0 0 0 0 0 0 0 0 0 0 0 0 0 0 0 0 0 0 0 0 0 0 0 0 0 0 0 0 0 0

[112] 0 0 0 0 0 0

$y2

[1] 3 5 3 5 2 3 4 3 2 2 3 3 2 4 4 5 3 3 0 2 0 5 0 0 3 6 2 0 6 0 0 0 0 0 0 0 0

[38] 2 0 0 0 0 0 0 6 3 4 4 5 8 4 7 3 9 5 0 0 4 2 0 6 0 0 0 0 7 0 3 3 3 3 4 3 3

[75] 5 3 0 2 5 5 3 4 0 0 1 4 3 0 5 3 2 3 4 2 5 4 6 6 3 2 2 6 0 5 6 6 2 3 3 2 0

[112] 3 0 4 0 10 1 4 3 5 5 4 2 3 2 5 4 2 4 3 7

$y3

[1] 3 6 9 3 2 3 4 3 2 2 3 2 3 4 3 4 6 6 NA NA NA NA NA NA NA NA NA NA NA NA NA NA NA NA NA NA NA

[38] NA NA NA NA NA NA NA NA NA NA NA NA NA NA NA NA NA NA NA NA NA NA NA NA NA NA NA NA NA NA 3 4 3 2 3 5 2

[75] 6 6 NA NA NA NA NA NA NA NA NA NA NA NA NA NA NA NA NA NA NA NA NA NA NA NA NA NA NA NA NA NA NA NA NA NA NA

[112] NA NA NA NA NA NA 3 4 6 7 3 3 3 3 6 3 4 6 3 7

$Works.pa

[1] FALSE FALSE FALSE FALSE FALSE FALSE FALSE FALSE FALSE FALSE FALSE TRUE TRUE TRUE TRUE TRUE TRUE TRUE

[19] FALSE FALSE FALSE FALSE FALSE FALSE FALSE FALSE FALSE FALSE FALSE FALSE FALSE FALSE FALSE FALSE FALSE FALSE

[37] FALSE FALSE FALSE FALSE FALSE TRUE TRUE TRUE TRUE TRUE TRUE TRUE TRUE TRUE TRUE TRUE TRUE TRUE

[55] TRUE TRUE TRUE TRUE TRUE TRUE TRUE TRUE TRUE TRUE TRUE TRUE TRUE FALSE FALSE FALSE FALSE FALSE

[73] TRUE TRUE TRUE TRUE FALSE FALSE FALSE FALSE FALSE FALSE FALSE FALSE FALSE FALSE FALSE FALSE FALSE FALSE

[91] FALSE FALSE FALSE TRUE TRUE TRUE TRUE TRUE TRUE TRUE TRUE TRUE TRUE TRUE TRUE TRUE TRUE TRUE

[109] TRUE TRUE TRUE TRUE TRUE TRUE TRUE TRUE TRUE FALSE FALSE FALSE FALSE FALSE TRUE TRUE TRUE TRUE

[127] TRUE TRUE TRUE TRUE TRUE

$nd

[1] -0.046986087 -0.172119279 -0.495898662 -0.076471164 -0.461983682 0.343698173 0.444035712 0.376037919

[9] 0.082668510 0.405127682 0.401171884 -0.659873707 0.021633728 -0.618488483 -1.571566745 -0.112520114

[17] 0.704307726 0.120405387 0.092107646 -0.540896059 0.078576650 0.317467457 0.172463007 -0.221496269

[25] 0.454525698 -0.427756812 -0.015681342 -0.560703284 -0.325538857 0.119128676 0.641280544 -0.006129832

[33] -0.114413075 0.217431915 0.559705719 0.424010784 0.173241169 0.227096005 0.336068384 -0.775189476

[41] -0.224533100 0.207607492 -0.260624158 0.351518723 -0.644206634 -0.644206634 -0.533155023 -0.618488483

[49] -1.316835593 -0.855643616 -1.000064349 -0.486347152 -0.217722000 -0.041372957 0.195374214 -0.186933390

[57] 0.158676998 0.364609269 -0.213224402 0.424265322 -0.112520114 0.821870970 0.362918128 0.258889308

[65] 0.317772155 -0.332804970 0.413738526 0.128427924 0.537006114 -0.225294748 0.217431915 -1.046994307

[73] 0.268898236 -0.122046016 -0.213224402 0.704307726 0.025638348 -0.427756812 0.123796554 -0.699257579

[81] 0.113564233 -0.113150429 -0.080635184 0.472154265 1.306091375 0.525321449 0.930942569 0.325639773

[89] -0.736007322 -0.736007322 1.167688713 0.352668589 -0.210988349 -0.373465431 0.364609269 0.138408736

[97] 0.122950581 0.222820313 0.445756098 0.203188212 -1.101302278 -0.846870407 -0.689163344 0.037003034

[105] -0.257397007 0.039445021 -0.113150429 0.246307330 0.507018599 -0.243874493 0.505689348 0.419410306

[113] 0.569941941 0.117849207 0.300462924 0.599440662 0.499670519

$nt.60

[1] 0.06219587 0.27714452 -0.15095917 0.27714452 0.21455770 0.38400273 -0.30065990 -0.03391667 0.47314643

[10] -0.85154048 -0.50861146 -0.85154048 0.47314643 -0.15095917 -0.30065990 -0.03391667 0.27714452 -0.15095917

[19] -0.03391667 -0.30065990 0.38400273 0.21455770 0.27714452 -0.03391667 0.38400273 0.43041469 0.61658635

[28] 0.47314643 0.43041469 -0.03391667 0.54962214 0.64718781 0.21455770 0.58414311 -0.03391667 0.43041469

[37] -0.30065990 0.33321641 -0.03391667 -0.15095917 -0.15095917 -0.30065990 0.14374053 -0.03391667 0.43041469

[46] 0.58414311 -0.30065990 -0.50861146 0.33321641 0.06219587 0.14374053 0.38400273 0.33321641 0.77855032

[55] 0.27714452 -0.85154048 -0.03391667 0.14374053 -0.50861146 -0.50861146 0.21455770 -0.85154048 0.43041469

[64] -0.15095917 -0.30065990 0.14374053 0.33321641 0.21455770 -0.15095917 0.38400273 -0.30065990 0.14374053

[73] 0.14374053 -0.15095917 -0.03391667 0.06219587 -2.03629738 0.06219587 0.64718781 0.61658635 0.43041469

[82] 0.27714452 -2.03629738 0.51273897 -2.03629738 -0.30065990 -0.03391667 0.38400273 0.06219587 -0.03391667

[91] 0.14374053 0.33321641 -0.30065990 0.21455770 -0.15095917 -0.15095917 -0.15095917 -0.30065990 0.21455770

[100] -0.03391667 0.21455770 -0.50861146 -0.50861146 0.21455770 0.38400273 0.33321641 0.51273897 -0.85154048

[109] 0.33321641 -0.85154048 0.38400273 0.06219587 0.06219587 0.06219587 -0.30065990 0.27714452 -0.85154048

$np.60

[1] 0.18014793 0.60127582 0.18014793 0.39311600 0.25881058 0.45144917 -0.46740662 0.18014793 0.18014793

[10] 0.09114091 -0.27925430 -0.13214867 0.50515683 -0.01134902 -0.27925430 -0.01134902 -0.27925430 -0.46740662

[19] 0.18014793 0.25881058 0.09114091 0.64466373 0.25881058 -0.01134902 -0.13214867 0.39311600 0.09114091

[28] 0.18014793 0.68544021 -0.27925430 0.45144917 0.25881058 0.32928489 -0.13214867 -0.01134902 0.39311600

[37] 0.18014793 0.09114091 0.09114091 0.09114091 0.60127582 -1.15978441 0.09114091 -1.15978441 0.39311600

[46] 0.25881058 -1.15978441 -0.13214867 0.39311600 -0.13214867 0.18014793 0.25881058 0.32928489 0.76029743

[55] -0.13214867 -0.72877187 -0.27925430 -0.13214867 -0.72877187 -0.01134902 -0.27925430 -0.72877187 0.25881058

[64] 0.25881058 -0.27925430 0.18014793 0.18014793 0.25881058 0.45144917 0.18014793 -0.27925430 0.39311600

[73] -0.01134902 0.09114091 -0.13214867 0.39311600 -0.72877187 0.45144917 0.32928489 0.39311600 0.09114091

[82] -0.27925430 -1.15978441 1.06821864 -1.15978441 0.18014793 0.09114091 -0.27925430 -0.72877187 0.32928489

[91] 0.09114091 -0.27925430 -0.46740662 0.45144917 0.18014793 -0.13214867 0.39311600 0.25881058 0.09114091

[100] 0.18014793 -0.01134902 0.68544021 0.09114091 -0.46740662 0.45144917 0.39311600 0.39311600 -0.72877187

[109] 0.18014793 -1.15978441 -0.01134902 -0.01134902 -0.01134902 -0.27925430 0.25881058 -0.13214867 -2.64885371

$YD

Y1 Y2 Y3 Y4

1 6 6 6 5

2 6 2 6 0

3 2 0 5 2

4 0 0 0 0

5 4 4 4 4

6 0 0 0 0

7 0 0 0 0

8 0 0 0 0

9 1 0 1 3

10 0 0 0 0

11 8 8 7 8

12 2 2 2 0

13 0 0 0 0

14 0 0 0 0

15 0 0 0 0

16 0 4 0 4

17 0 2 2 2

18 0 0 0 0

19 2 2 2 2

20 0 0 0 0

21 0 0 0 0

22 0 0 0 0

23 0 0 0 0

24 0 0 0 0

25 0 0 0 0

26 0 0 0 0

27 0 0 0 0

28 2 2 2 2

29 0 0 0 0
