# Supplementary figures and images for: Demographic Effects of Habitat Restoration for the Grey-Crowned Babbler Pomatostomus temporalis, in Victoria, Australia
Source: PLoS One. 2015 Jul 15;10(7):e0130153. doi: 10.1371/journal.pone.0130153 (PMC4503698; doi:10.1371/journal.pone.0130153)

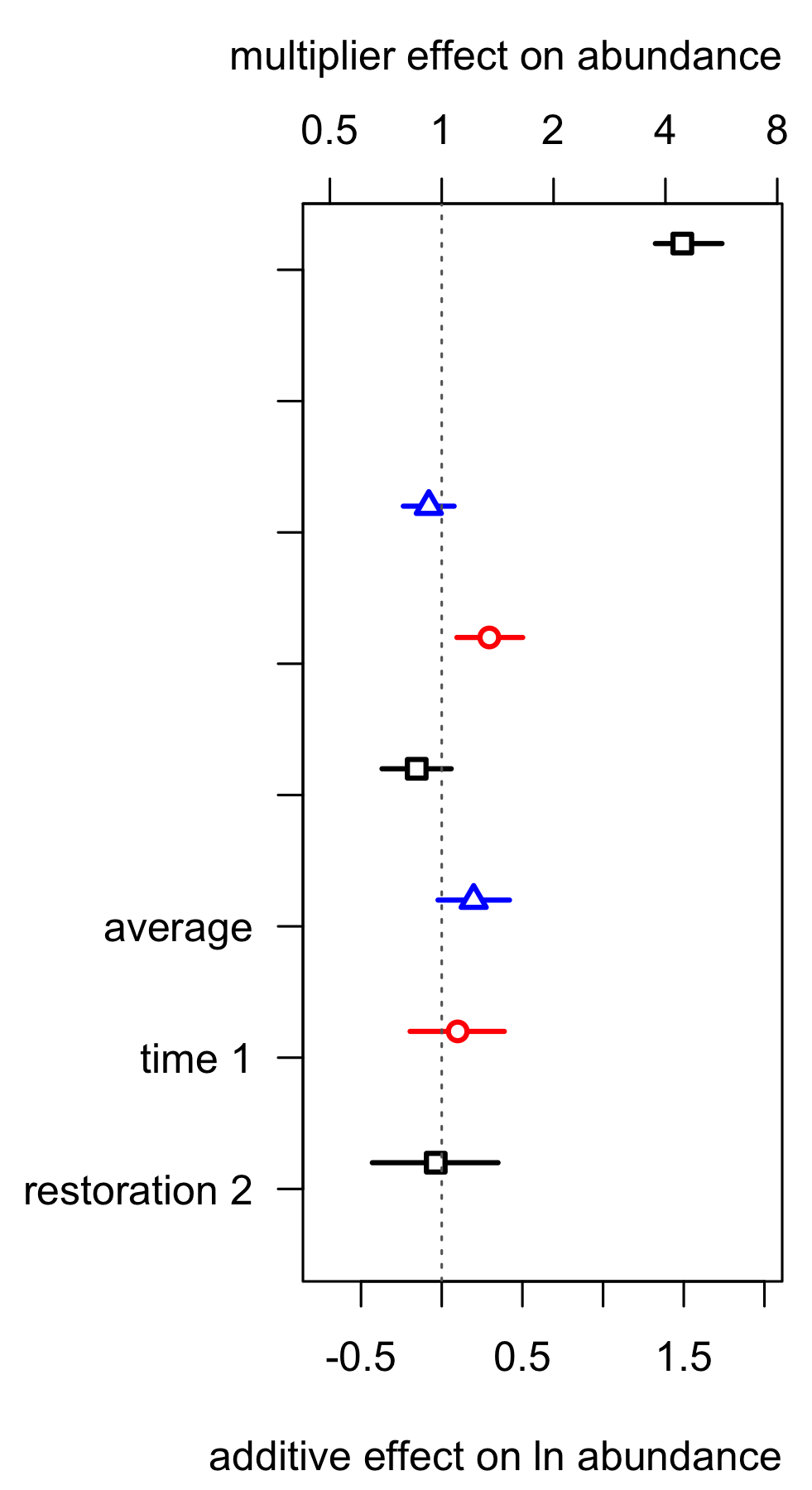

Supplement: S1 Fig — Symbols represent point estimates as the posterior median (and 95% credible intervals represented as lines). Alternative models: M2, which included a site random effect (blue triangles); M6, which in addition incorporated the linear model for occupancy for Set 2 in 2008 (red circles); and, M4 which included the possibility of difference between restored and unrestored sites in 1995 (black squares). The x-axis presents the effect size, which is on a logit scale, the second x-axis, above the graph, presents the effects transformed back into odds ratios (or odds for the intercept). The parameter estimates for the various effects represent the additive change to the mean probability of occupancy resulting from the observed range for the particular factor. Interpretation of effects on the raw scale is as the multiplicative change to the raw mean probability of occupancy. (TIF) [file pone.0130153.s003.tif]

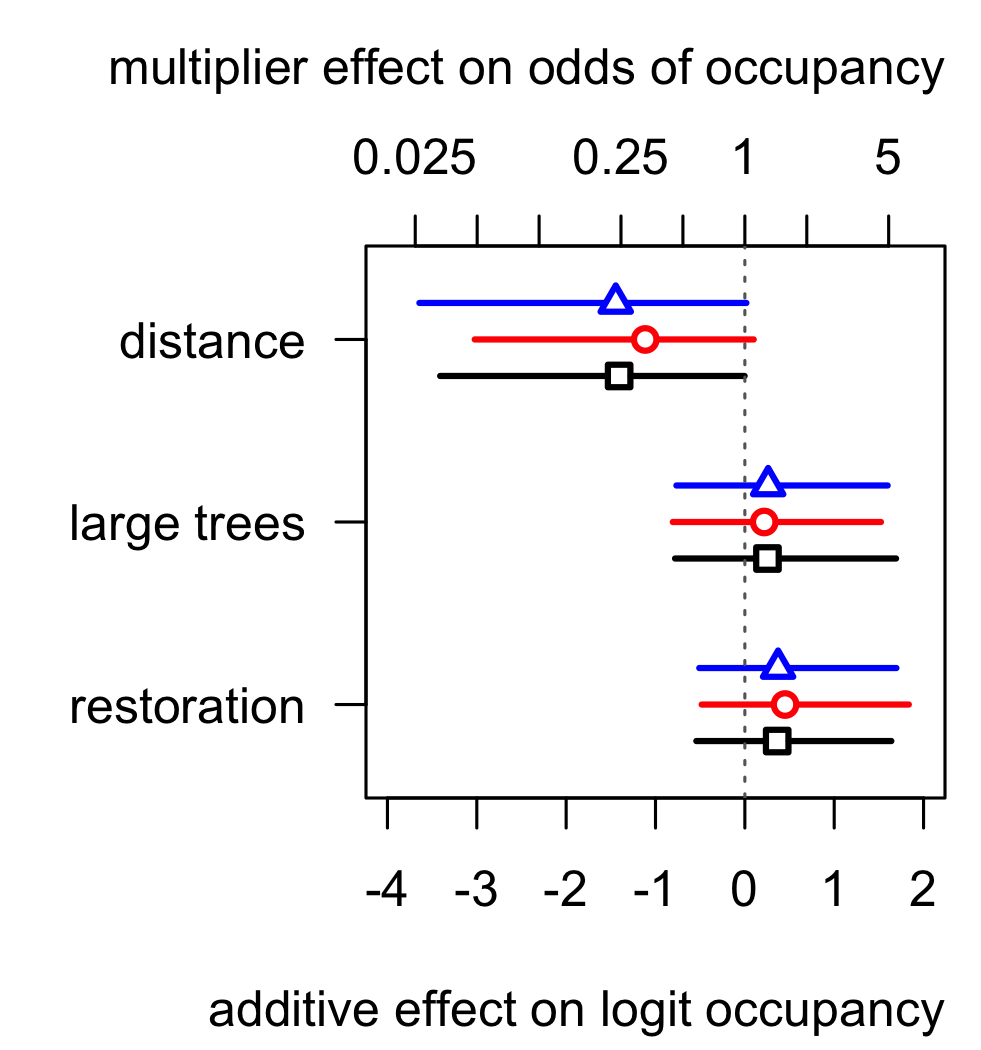

Supplement: S2 Fig — Symbols represent point estimates as the posterior median (and 95% credible intervals represented as lines). Alternative models: M2, which included a site random effect (blue triangles); M6, which in addition incorporated the linear model for occupancy for Set 2 in 2008 (red circles); and, M4 which included the possibility of difference between restored and unrestored sites in 1995 (black squares). The x-axis presents the effect size, which is on a log scale, the second x-axis, above the graph, presents the effects transformed back into raw numbers. The ‘average’ is the intercept and is the mean group size in 1995 for the average site, at the average distance from nearest group and with the average density of large trees. The parameter estimates for the various effects represent the additive change to the log(mean group size) resulting from the observed range for the particular factor. Interpretation of effects on the raw scale is as the multiplicative change to the raw mean group size. (TIF) [file pone.0130153.s004.tif]

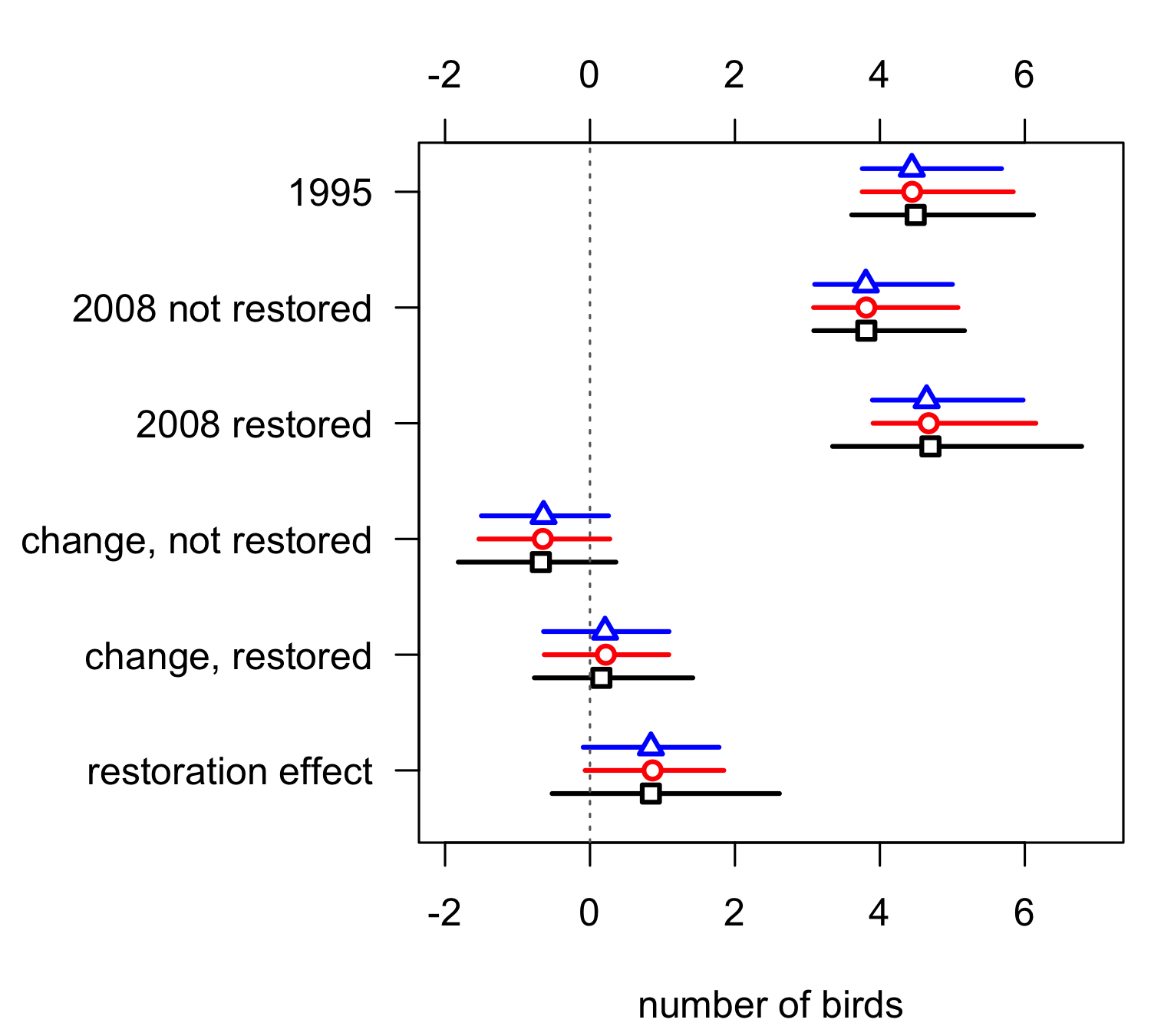

Supplement: S3 Fig — Alternative models: M2, which included a site random effect (blue triangles); M6, which in addition incorporated the linear model for occupancy for Set 2 in 2008 (red circles); and, M4 which included the possibility of difference between restored and unrestored sites in 1995 (black squares). (TIF) [file pone.0130153.s005.tif]
