# Supplementary material for: Demographic Effects of Habitat Restoration for the Grey-Crowned Babbler Pomatostomus temporalis, in Victoria, Australia
Source: PLoS One. 2015 Jul 15;10(7):e0130153. doi: 10.1371/journal.pone.0130153 (PMC4503698; doi:10.1371/journal.pone.0130153)
Supplement: S1 Table — (DOCX) [file pone.0130153.s006.docx]

**Table S1. Comparison of alternative models with posterior mean and SD of deviance.**

| Model | Deviance (+SD) |
| --- | --- |
| M2 site random effect | 478.8 + 87.1 |
| M6 site random effect & linear model for occupancy of Set 2 | 484.4 + 91.1 |
| M4 site random effect & start differences between unrestored and restored sites | 485.1 + 92.8 |
| M5 base model & linear model for occupancy of Set 2 | 488.9 + 94.1 |
| M3 start differences between unrestored and restored sites | 489.7 + 95.9 |
| M7 start differences between unrestored and restored sites & linear model for occupancy of Set 2 | 489.9 + 93.7 |
| M1 base model | 493.2 + 96.8 |
